# Vicious Vanco: Vancomycin-Induced Cutaneous Small-Vessel Vasculitis in a Patient With End-Stage Renal Disease

**DOI:** 10.7759/cureus.101537

**Published:** 2026-01-14

**Authors:** Michelle Carrasquel, Khaleel Quasem, Taylor Patterson, Eglal Samir, Chris Farnum

**Affiliations:** 1 Internal Medicine, McLaren Greater Lansing Hospital, Lansing, USA; 2 Internal Medicine, Michigan State University, Lansing, USA; 3 Medicine, McLaren Greater Lansing Hospital, Lansing, USA; 4 Infectious Disease, Michigan State University, Lansing, USA

**Keywords:** antibiotic reaction, drug induce, hemodialysis complications, small vessel vasculitis, vancomycin hypersensitivity

## Abstract

Vancomycin is a first-line antibiotic used for severe gram-positive infections, but it may rarely trigger immune-mediated small-vessel vasculitis. We report a case of a 43-year-old man with end-stage renal disease on home hemodialysis who developed palpable purpuric skin lesions three days after starting vancomycin for L5-S1 discitis and osteomyelitis. Over the following days, the eruption progressed into symmetric, non-blanching purpuric plaques involving the upper and lower extremities by day 6 of therapy. Laboratory evaluation demonstrated progressive thrombocytopenia, an increase in eosinophil counts within the normal reference range, and elevated inflammatory markers, which declined after vancomycin cessation. Vancomycin was discontinued, and antimicrobial therapy was transitioned to daptomycin, resulting in rapid clinical improvement within 48 hours and near-complete resolution by day 12. This case emphasizes the importance of early recognition of vancomycin-induced leukocytoclastic vasculitis, particularly in patients with renal impairment who may experience an accelerated onset. Prompt discontinuation of the offending agent typically results in rapid recovery without the need for systemic corticosteroids.

## Introduction

Vancomycin remains essential for the treatment of serious gram-positive infections, including methicillin-resistant *Staphylococcus aureus* bacteremia, endocarditis, osteomyelitis, and discitis. Although generally well tolerated, vancomycin is associated with a spectrum of hypersensitivity reactions ranging from benign exanthems and infusion-related erythema to severe immune-mediated conditions such as Stevens-Johnson syndrome and vasculitis. Leukocytoclastic vasculitis is a small-vessel inflammatory disorder caused by immune-complex deposition in post-capillary venules, leading to complement activation and neutrophilic infiltration.

Drug-induced leukocytoclastic vasculitis accounts for approximately 10-20% of all cases of cutaneous leukocytoclastic vasculitis reported in the literature. While beta-lactam antibiotics and sulfonamides are more frequently implicated, vancomycin-associated leukocytoclastic vasculitis remains an uncommon but well-documented adverse reaction. The latency period between drug exposure and symptom onset is typically reported as five to 10 days; however, earlier onset has been described in patients with impaired renal clearance, likely due to prolonged drug exposure and altered immune complex handling.

This report describes the clinical course, laboratory trends, and treatment response of a patient with end-stage renal disease who developed rapidly progressive vancomycin-induced leukocytoclastic vasculitis, illustrating a classic presentation with prompt resolution following discontinuation of the offending agent.

## Case presentation

A 43-year-old man with Alport syndrome-related end-stage renal disease on home hemodialysis five times weekly presented with worsening low-back pain, dyspnea requiring supplemental oxygen, intermittent confusion, and generalized malaise. Eleven days earlier, he had been hospitalized for a tunneled dialysis catheter-related bloodstream infection and discharged on cefazolin following catheter exchange. Despite completing several days of cefazolin therapy, his symptoms persisted, prompting re-evaluation.

Magnetic resonance imaging demonstrated discitis and osteomyelitis at the L5-S1 level with small associated right iliopsoas and left sacroiliac fluid collections. Given concern for ongoing vertebral infection in the setting of recent catheter-related bacteremia, the patient was admitted and empirically started on intravenous vancomycin for broad coverage of gram-positive organisms, including methicillin-resistant *Staphylococcus aureus*, which is among the most common etiologies of catheter-associated bloodstream infections and vertebral osteomyelitis in hemodialysis patients. Blood cultures obtained during the current admission did not yield persistent growth, and prior cultures had not definitively identified the organism at the time of vancomycin initiation; thus, vancomycin was selected empirically rather than to target a confirmed *Bacillus* species.

**Figure 1:**
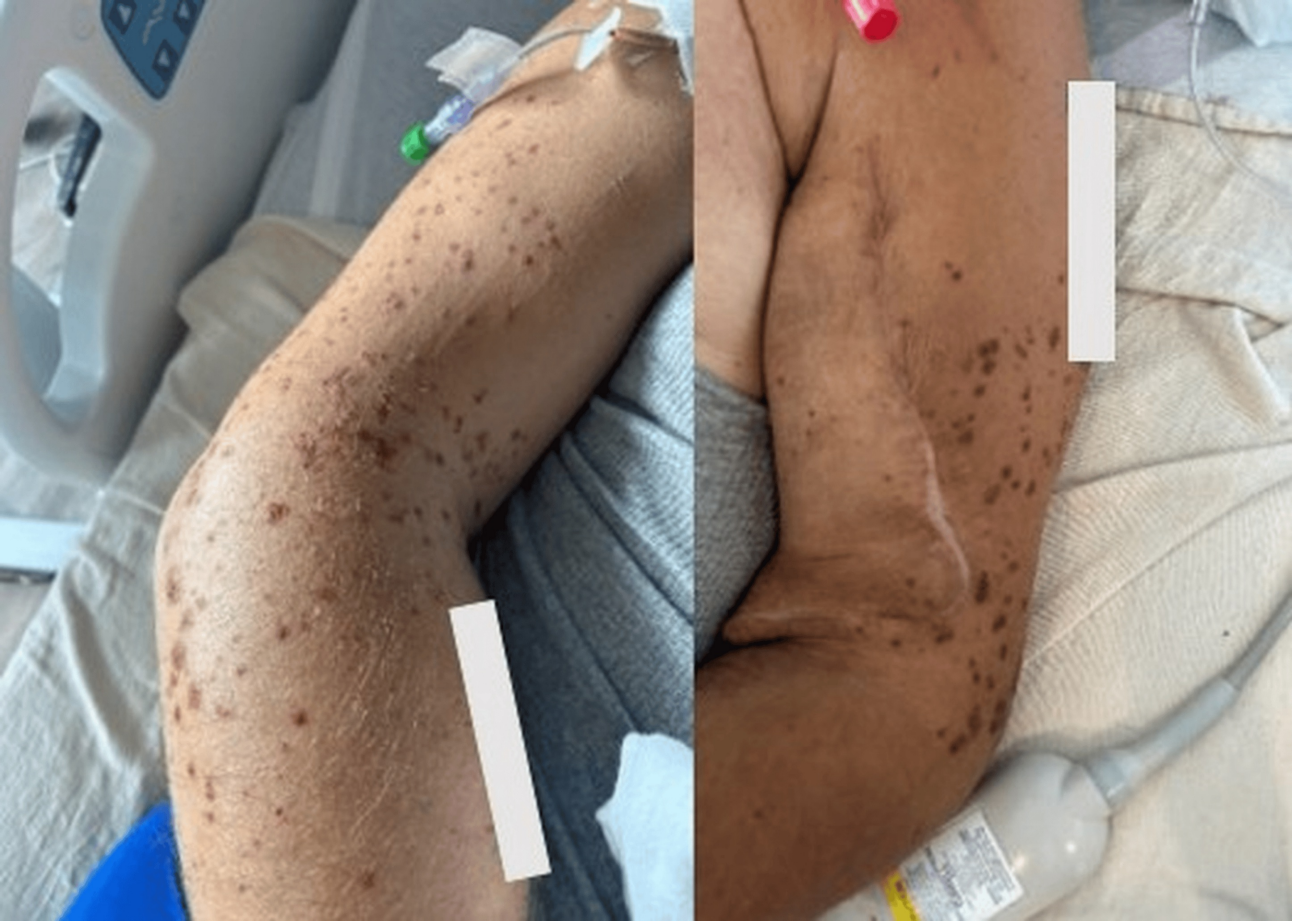
Hospital day 3 (three days after initiation of vancomycin) Initial onset of palpable, non-blanching purpuric papules involving the upper extremities. Lesions are discrete, erythematous-to-violaceous, and palpable, consistent with early cutaneous small-vessel vasculitis temporally associated with vancomycin exposure.

Baseline laboratory testing demonstrated chronic anemia and elevated inflammatory markers, with C-reactive protein increasing from 4.8 to 7.2 milligrams per deciliter during the first 48 hours of vancomycin exposure (Table 1). Absolute eosinophil counts remained within the normal reference range throughout hospitalization, although a relative increase from baseline was observed early in the course. Approximately three days after initiating vancomycin, the patient developed new, palpable, non-blanching purpuric papules on both the upper and lower extremities. Over the subsequent three days, the eruption progressed, and by day 6 of vancomycin therapy, the lesions had evolved into symmetric erythematous-to-violaceous palpable purpuric plaques consistent with a small-vessel vasculitic process, without mucosal involvement or systemic vasculitic manifestations (Figure 1).

**Table 1:** Laboratory results during hospital course CRP: C-reactive protein, mg/dL: milligrams per deciliter, g/dL: grams per deciliter, ×10⁹/L: billion cells per liter

Hospital day	Hemoglobin (g/dL)	Platelets (×10⁹/L)	White blood cells (×10⁹/L)	Eosinophils (%)	Eosinophils (×10⁹/L)	Creatinine (mg/dL)	C-reactive protein (mg/dL)	Notable events
Reference values	13.5–17.5	150–450	4.0–11.0	0–6	0.00–0.50	0.6–1.3	<1.0	–
Day 1	7.5	232	7.07	2.4	0.16	8.5	4.8	Vancomycin started
Day 2	7.7	235	–	–	–	7.6	6.2	Increase in inflammatory markers
Day 3	8	217	–	4.1	0.29	8.5	7.2	Pre-eruption inflammatory peak
Day 4	8	192	–	–	–	6.9	–	Vancomycin discontinued
Day 5	7.8	196	–	–	–	8.2	–	Rash progressing
Day 6	7.6	168	–	–	–	6.2	–	Early purpura evolving
Day 7	8.5	162	5.84	1.9	0.11	–	3.4	Rash visible; daptomycin initiated
Day 11	8.6	114	7.12	2.5	0.18	8.6	2.7	Rash improving
Day 12	8.4	109	7.02	2.4	0.17	8.6	–	Near-complete resolution

Concurrent laboratory trends demonstrated progressive thrombocytopenia, with platelet counts declining from 232 ×10⁹ per liter at baseline to 162 ×10⁹ per liter at the time of rash evolution and further decreasing to a nadir of 109 ×10⁹ per liter later in the hospital course (Table 1). Given the temporal association with vancomycin exposure, absence of hemolysis, stable renal parameters, and lack of neurologic or gastrointestinal involvement, alternative diagnoses such as thrombotic microangiopathy, infection-related vasculitis, or systemic immune-mediated vasculitis were considered less likely.

Cefazolin was deemed an unlikely contributor to the cutaneous findings, as the patient had been exposed to this agent for more than one week prior to rash onset without dermatologic manifestations, and the eruption developed only after initiation of vancomycin, with clear progression during continued vancomycin exposure and improvement following its discontinuation.

**Figure 2:**
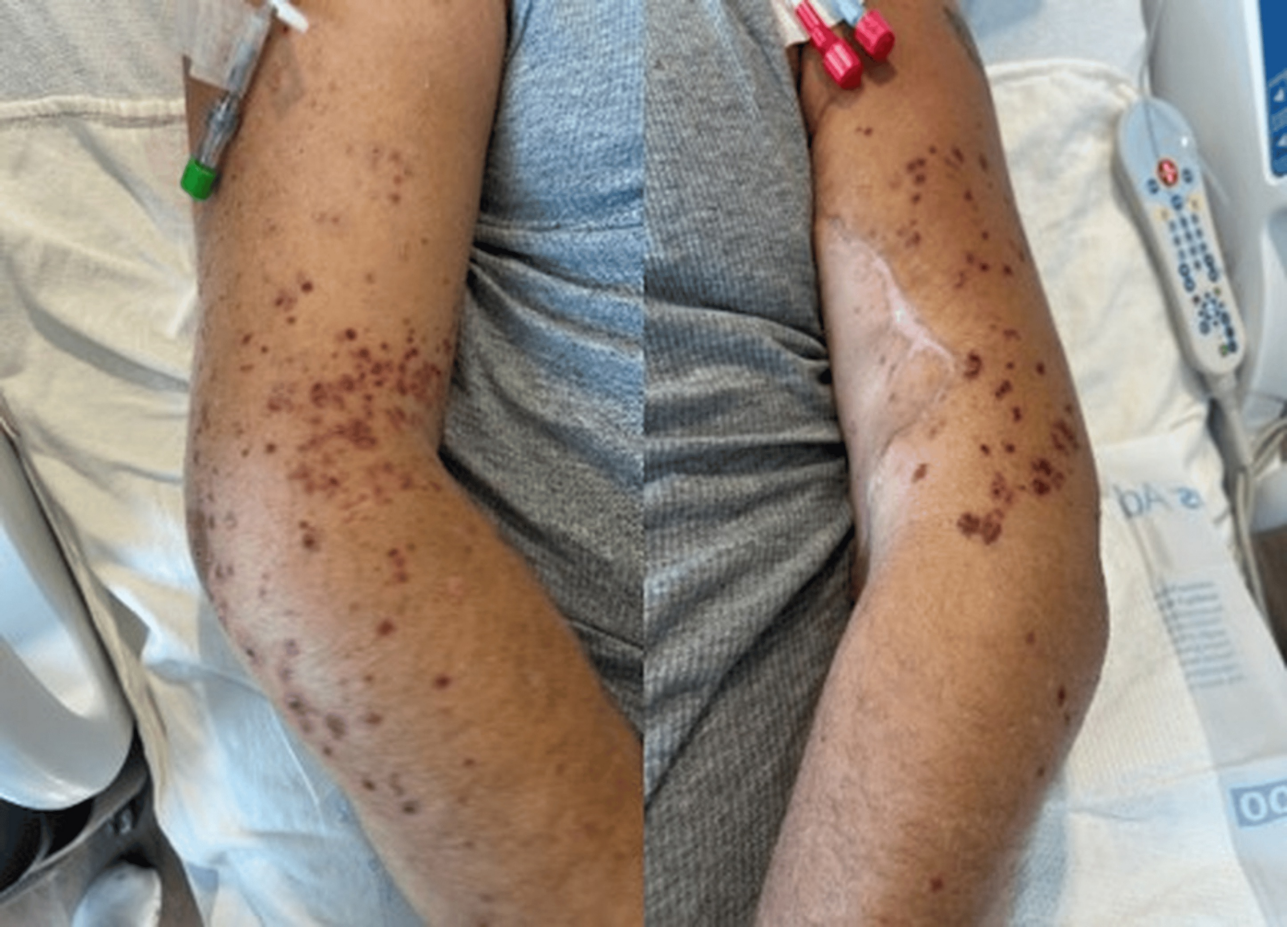
Hospital day 6 (48 hours after vancomycin discontinuation on hospital day 4) Early clinical improvement with flattening, decreased palpability, and fading of previously purpuric lesions 48 hours after cessation of vancomycin.

**Figure 3:**
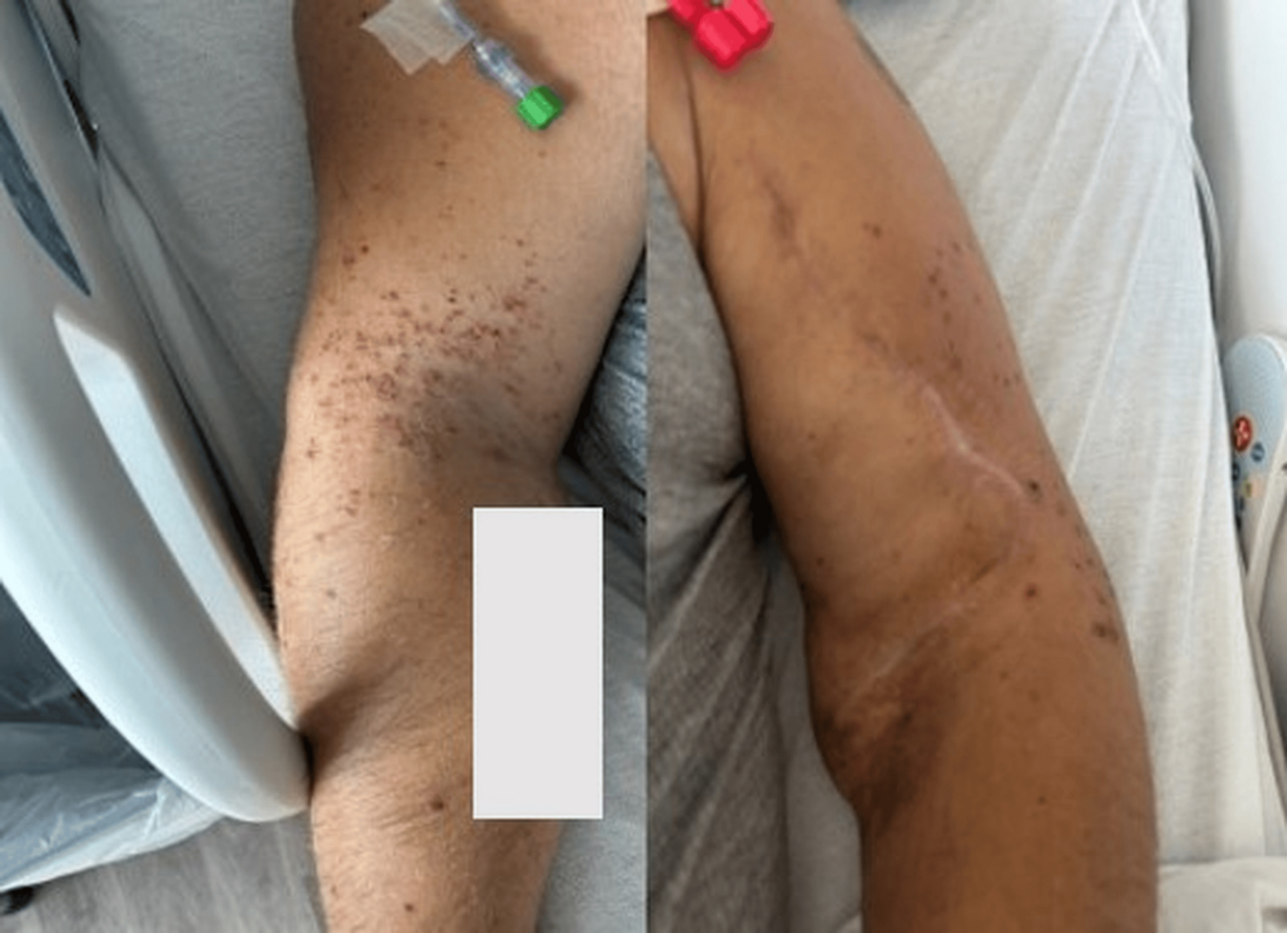
Hospital day 12 (8 days after vancomycin discontinuation on hospital day 4) Near-complete resolution of cutaneous lesions, with residual post-inflammatory hyperpigmentation and absence of active purpura, was observed 8 days after vancomycin discontinuation.

Based on the characteristic morphology, symmetric distribution, and close temporal relationship between vancomycin exposure and rash onset and progression, vancomycin-induced cutaneous small-vessel vasculitis was strongly suspected. Vancomycin was discontinued on hospital day 4, and antimicrobial therapy was transitioned to daptomycin for continued coverage of gram-positive organisms. A skin biopsy was offered but declined due to rapid clinical improvement. Within 48 hours of vancomycin cessation, the purpuric lesions flattened and faded significantly (Figure 2), with improvements in inflammatory markers and stabilization of platelet counts (Table 1). By hospital day 12 (eight days after vancomycin discontinuation), the rash had nearly resolved (Figure 3), and no systemic organ involvement developed during hospitalization. The patient completed the remainder of his antimicrobial course with daptomycin without complication and was discharged home with outpatient follow-up. Application of the Naranjo Adverse Drug Reaction Probability Scale yielded a total score of 7, consistent with a probable adverse drug reaction. Positive scoring parameters included a clear temporal relationship between vancomycin initiation and symptom onset, objective clinical evidence of vasculitis, improvement following drug discontinuation, absence of alternative etiologies, and prior published reports of vancomycin-induced cutaneous small-vessel vasculitis.

## Discussion

Vancomycin-induced cutaneous small-vessel vasculitis is a rare immune-complex-mediated hypersensitivity reaction characterized by palpable, non-blanching purpura resulting from leukocytoclastic inflammation of post-capillary venules. Drug-induced vasculitis accounts for approximately 10% to 20% of all cases of cutaneous small-vessel vasculitis. Although beta-lactam antibiotics and sulfonamides are more commonly implicated, vancomycin remains an uncommon but consistently reported trigger in the medical literature [1-7].

Symptom onset typically occurs within five to 10 days of exposure; however, earlier onset has been described in patients with impaired kidney function due to reduced drug clearance and prolonged antigen exposure. This phenomenon has been documented in pharmacologic and immunologic studies involving renally cleared medications [8-10]. In the present case, the appearance of palpable purpura three days after vancomycin initiation is consistent with early-onset vasculitis in the setting of end-stage renal disease requiring hemodialysis (Figure 1).

The differential diagnosis of acute purpura in hospitalized patients is broad. It includes septic emboli, infection-related vasculitides, allergic drug eruptions, immunoglobulin A vasculitides, and hematologic emergencies such as thrombotic microangiopathy. Progressive thrombocytopenia raised the consideration of microangiopathic processes. However, although creatinine values fluctuated during hospitalization, these changes were consistent with baseline variability in a patient with end-stage renal disease on chronic hemodialysis and did not represent acute kidney injury. There was no laboratory evidence of hemolysis, no schistocytes were reported, and no neurologic or gastrointestinal manifestations developed, making thrombotic microangiopathy unlikely. Severe hypertension-induced thrombotic microangiopathy can mimic small-vessel vasculitis, as described by Valdez-Thomas et al., and must be considered carefully in such presentations; however, the overall clinical context and temporal relationship to vancomycin exposure supported a drug-induced vasculitic process rather than a primary microangiopathy [4,5].

Microbiologic evaluation during admission included repeat blood cultures, which did not demonstrate persistent growth, and there was no microbiologic evidence of ongoing bacteremia or infectious vasculitis. No bone biopsy was performed, and antimicrobial therapy was guided by clinical, radiographic, and laboratory findings rather than organism isolation. The absence of positive cultures and the rapid cutaneous improvement after vancomycin discontinuation further supported a noninfectious etiology for the rash.

Management of vancomycin-induced cutaneous small-vessel vasculitis centers on prompt discontinuation of the offending agent. Vancomycin desensitization was not pursued in this case, as effective alternative antimicrobial options were available and the hypersensitivity reaction resolved rapidly with drug withdrawal. Consistent with prior reports, the patient demonstrated marked improvement within 48 hours after discontinuation of vancomycin (Figure 2) and near-complete resolution eight days after discontinuation of vancomycin (Figure 3) without the need for systemic corticosteroids [1-3].

For patients with vertebral osteomyelitis who cannot tolerate vancomycin, alternative treatment options include daptomycin, linezolid, ceftaroline, and, in select cases, long-acting lipoglycopeptides such as dalbavancin or oritavancin, depending on organism susceptibility, renal function, and treatment duration requirements. In this case, daptomycin was selected for its efficacy against gram-positive organisms, favorable safety profile in patients with renal impairment, and lack of known association with immune-mediated vasculitis, and it was well tolerated without recurrence of cutaneous findings.

This case highlights the importance of maintaining a high index of suspicion for vasculitic eruptions in patients receiving vancomycin, particularly those with end-stage renal disease. Early recognition, exclusion of alternative diagnoses, and timely discontinuation of the offending agent are essential to prevent systemic involvement and ensure rapid clinical recovery.

## Conclusions

Vancomycin-induced cutaneous small-vessel vasculitis is an uncommon but clinically important hypersensitivity reaction that should be promptly recognized, especially in patients with renal impairment who may experience an accelerated onset. The characteristic presentation of new palpable, non-blanching purpura, such as seen in this patient, in close temporal association with vancomycin initiation, should raise immediate concern for drug-induced vasculitis. Early medication withdrawal remains the cornerstone of management and typically results in rapid improvement, often within 48-72 hours, without the need for systemic corticosteroids. This case underscores the importance of maintaining a high index of suspicion for vasculitic eruptions during vancomycin therapy, ensuring timely discontinuation, preventing systemic involvement, and allowing for safe transition to alternative antimicrobial agents such as daptomycin.
